# Production of Graphene Membranes from Rice Husk Biomass Waste for Improved Desalination

**DOI:** 10.3390/nano14020224

**Published:** 2024-01-19

**Authors:** Makpal Seitzhanova, Seitkhan Azat, Mukhtar Yeleuov, Azamat Taurbekov, Zulkhair Mansurov, Erlan Doszhanov, Ronny Berndtsson

**Affiliations:** 1 Faculty of Chemistry and Chemical Technology, Department of Chemical Physics and Materials Science, Al-Farabi Kazakh National University, Al-Farabi Ave. 71, Almaty 050059, Kazakhstan; seitzhanova.makpal@kaznu.kz (M.S.); zmansurov@kaznu.kz (Z.M.); 2 Laboratory of Engineering Profile, Satbayev University, Satbayev Str. 22a, Almaty 050013, Kazakhstan; 3 Institute of Combustion Problems, Bogenbay Batyr Str. 1721, Almaty 050012, Kazakhstan; mukhtar.yu@gmail.com (M.Y.); a.taurbek@gmail.com (A.T.); yerlan.doszhanov@gmail.com (E.D.); 4 Division of Water Resources Engineering, Centre for Advanced Middle Eastern Studies, Lund University, P.O. Box 118, SE-22100 Lund, Sweden

**Keywords:** graphene membranes, desalination, biomass waste, rice husk, water treatment, nanotechnology, green technology

## Abstract

Inexpensive and efficient desalination is becoming increasingly important due to dwindling freshwater resources in view of climate change and population increase. Improving desalination techniques of brackish water using graphene-based materials has the possibility to revolutionize freshwater production and treatment. At the same time, graphene matter can be cheaply mass-produced from biowaste materials. In view of this, graphene material was obtained from a four-step production approach starting from rice husk (RH), including pre-carbonation, desilication, chemical activation, and exfoliation. The results showed that the produced samples contained a mixture of graphene layers and amorphous carbon. The activation ratio of 1:5 for carbonized RH and potassium hydroxide (KOH), respectively, provided higher graphene content than the 1:4 ratio of the same components, while the number of active layers remained unaffected. Further treatment with H_2_O_2_ did not affect the graphene content and exfoliation of the amorphous carbon. Preparation of the graphene material by the NIPS technique and vacuum filtration displayed different physicochemical characteristics of the obtained membranes. However, the membranes’ main desalination function might be related more to adsorption rather than size exclusion. In any case, the desalination properties of the different graphene material types were tested on 35 g/L saltwater samples containing NaCl, KCl, MgCl_2_, CaSO_4_, and MgSO_4_. The produced graphene materials efficiently reduced the salt content by up to 95%. Especially for the major constituent NaCl, the removal efficiency was high.

## 1. Introduction

Access to safe water is one of the most important U.N. Sustainable Development Goals [1,2,3]. In pace with population growth and climate change, available surface and groundwater tend to become increasingly saline [4,5,6,7]. Increasing salinity threatens water security, global economic growth, and political stability [8]. For this reason, it is important to find inexpensive and improved methods for desalination of polluted and saline surface water and groundwater [9]. The salinity of surface water and groundwater can generally stem from two main mechanisms, namely land surface processes, such as irrigation and drainage, and saltwater intrusion to the groundwater from the sea [10].

Using biowaste for desalinization purposes can be a way to introduce economic green technologies in water treatment. For this purpose, available local biowaste could be used as a carbon source [11,12]. In this sense, rice husk (RH) is a substantial part of the produced rice crop [11,12,13]. RH is the outer covering of rice grains and one of the most widely available agricultural wastes in many rice-producing countries. The global annual RH biowaste production has been estimated to be about 160 million metric tons [14]. Converting some of this biowaste production into green products can increase the value chain and, at the same time, bring significant benefits to the environment [11].

Graphene is a one-atom-thick sheet of hybridized sp^2^ carbon atoms arranged in a cellular lattice with some outstanding properties. Graphene can, e.g., be produced from RH [11] and used for desalination processes [15,16,17]. However, graphene use has multifunctional applications ranging from energy and environmental protection to healthcare [18]. Graphene can be synthesized by numerous chemical or mechanical methods [19], but new methods for producing graphene using unconventional and inexpensive precursors are emerging. In this context, renewable and sustainable resources in the form of biowaste appear as especially important candidates for graphene precursors [19]. Along this line, the first attempts to obtain graphene-related materials from RH have recently been proposed [20,21,22,23].

Using RH directly for the production and synthesis of value-added materials is a feasible strategy to mitigate the recycling problem and reduce waste treatment costs for society and the environment [20,24]. In view of the above, graphene nanomaterial was synthesized from RH, and the morphological and chemical–physical properties were evaluated. The resulting graphene nanomaterial was applied in desalination experiments. Traditional reverse osmosis uses membranes of low water permeability [15]. Thus, it needs large amounts of energy in the desalination process [25]. Consequently, the use of graphene nanomaterial can revolutionize water treatment by introducing dynamic nanopores on the surface of the graphene and minimizing energy needs. RH represents a local biomaterial that can be used as a source for graphene synthesis in many regions. However, there are still only a handful of studies on the use of RH for desalination. Similarly, there is a need to further decipher not only the adsorption of salts but also further experiments on graphene’s possible sieving properties [12]. In view of this, the present study is innovative since it followed the entire line of nanomaterial production from RH to the desalination results for different types of salt. Thus, the objective of this study was to find improved methods for desalination and evaluate the production process of graphene nanomaterial for this purpose.

## 2. Materials and Methods

### 2.1. Materials

Potassium hydroxide and sodium hydroxide were purchased from Labhimprom Ltd. (Chemical Analytical Trading Company, Almaty, Kazakhstan) (TU 24363-80). RH was delivered from Bakanas village, the district center of Balkhash, Almaty region, Kazakhstan. Graphite was delivered from Russia. Hydrogen peroxide (37%) was purchased from Laborpharma Ltd. (Trade and Production Company, Almaty, Kazakhstan) (OST 301-02-206-99). The different salts, such as NaCl, KCl, MgCl_2_, CaSO_4_, and MgSO_4_, were purchased from Group of companies “ALTEY”, LLP “Laborpharma” (st. Amangeldy 52, Almaty, Kazakhstan) (GOST 4233-77) as well.

### 2.2. Graphene Synthesis

Carbonaceous material containing graphene was synthesized from RH using a top-down method following a four-step methodology: pre-carbonation, desilication, chemical activation, and exfoliation. The RH was first washed with distilled water several times to remove impurities and then dried at 383 K for 1 h. The RH was then pre-carbonated for 45 min in a rotating reactor under argon (Ar) injection at a temperature in the range of 523–573 K at a gas flow rate of 5 cm^3^/min. The resulting pre-carbonated RH (CRH) was desilicated by treating 60 g of CRH with 3 L of 1 M NaOH solution at 353 K for 3 h. The suspension was decanted to remove sodium silicate surfactant, washed to neutral pH, and oven-dried (2 h at 383 K). The desilicated sample was mixed with KOH using two ratios of carbon and KOH (1:4 and 1:5). The mixtures were compacted in an iron crucible and annealed at 1123 K for 2 h in an Ar atmosphere. After activation treatment, the resulting samples, labeled as Gr(1/4) and Gr(1/5), respectively, were washed with distilled water to a neutral content and dried at 373 K for 24 h. The same process was carried out on Gr(1/4) exfoliation to remove amorphous carbon by treating the material with a solution of hydrogen peroxide (H_2_O_2_, 37% *v*/*v*) for 48 h [24,26], then it was washed and dried. The product yield was ~3% by weight.

#### Graphene-Polymer Synthesis

**Figure 1:**
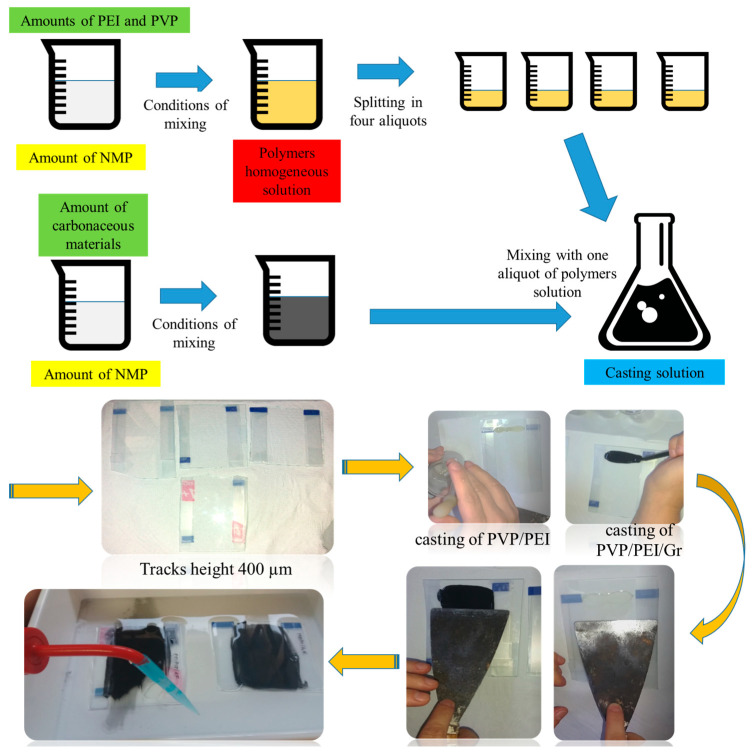
Schematic of experimental methodology for obtaining the graphene nanomaterials.

Polyethylenimine- (PEI) (mw ~25,000, Sigma Aldrich from Group of companies “ALTEY”, LLP “Laborpharma” (st. Amangeldy 52, Almaty, Kazakhstan)) or polyvinylpyrrolidone (PVP) (mw ~360,000, Sigma Aldrich from Group of companies “ALTEY”, LLP “Laborpharma” (st. Amangeldy 52, Almaty, Kazakhstan))-modified carbon nanomaterials were prepared by non-immersion precipitation system (NIPS technology) (Figure 1) [27]. Carbon black (CB) was used as a carbon model for comparison. First, 0.41 g of PEI and 1.95 mL of N-methyl-2-pyrrolidone (NMP) were added to a heat-resistant glass beaker and heated at about 50 °C by stirring for 18 h to obtain a homogeneous polymer suspension. Then, 0.1 g of the graphene sample was dispersed in a small amount of NMP (1 mL) and sonicated for 30 min to obtain a homogeneous dispersion and added to the PEI suspension.

Subsequently, 0.1 g of polyvinylpyrrolidone (PVP) was added to the PEI/carbon dispersion and stirred gently for 1 h until a homogeneous suspension was obtained. The resulting suspension was degassed by ultrasonication to remove trapped air bubbles. A homogeneous casting suspension was poured onto a glass plate (bringing the thickness to 400 microns) and immersed in a coagulation bath containing water as a non-solvent. The resulting membrane was kept in a coagulation bath for 24 h to ensure complete phase inversion.

Desalination of saline liquids was tested using a conventional filtration system at room temperature. The initial solution was loaded into a mixing cell with an effective membrane area of 38 mm^2^ and a loading tank with a capacity of 1000 mL. The total volume of the initial solution was 200 mL, considering both the volume of the mixing cell and the supply container. The filtration pressure was provided and regulated by a pump. Each of the fabricated graphene nanomaterial types was used for filtration tests. A steady flow of distilled water was measured and compared between different membrane samples to verify the repeatability of the graphene nanomaterial desalination properties.

### 2.3. Measurements

Scanning electron microscopy was performed on Au powder-coated samples using a field emission SEM (Nova NanoSEM 450 FEI/Termofisher at «National nanotechnology laboratory of open type» Almaty, Kazakhstan) at 3.00 kV in high vacuum mode, using an Everhart Thornley detector (ETD) and through a lens detector (TLD) for detailed micrographs and elemental microanalysis (EDX) at a voltage of 15.00 kV. The carbon, hydrogen, and nitrogen contents of the samples were measured with a 628 LECO elemental analyzer in accordance with ASTM E870 [28]. Material analysis by infrared spectroscopy (FTIR) was carried out on dispersions of solid samples obtained by mixing and grinding powdered materials (0.5–0.8 wt.%) with KBr. The granules were obtained by a pressure of 10 tons for 10 min. IR spectra in the range of 400–3400 cm^−1^ were obtained in transmission mode using a Nicolet 5700 spectrophotometer. The electrical conductivity of the beads was measured in four-contact geometry (low currents were recorded using a Keithley model 6485 picoammeter). Raman spectroscopy was performed on a confocal Raman microscope (Jasco, NRS-3100 at «National nanotechnology laboratory of open type» Almaty, Kazakhstan). A water-cooled 514 nm Ar+ laser line with a power of 4 MW per sample was introduced into an Olympus integrated microscope and focused to a spot diameter of approximately 2 μm with a 100× objective. A holographic notch filter was used to deflect the exciting laser line. Raman scattering was recorded using a Peltier cooled CCD photon detector with a resolution of 1024 × 128 pixels (Andor DU401BVI at «National nanotechnology laboratory of open type» Almaty, Kazakhstan). Raman measurements were repeated at least three times to ensure reproducibility. Cyclohexane was used for calibration.

Membrane tests for desalination were carried out on an atomic absorption flame emission spectrophotometer (Shimadzu AA-6200 at the Center for Physicochemical Research and Analysis (Almaty, Kazakhstan). The atomic absorption flame emission spectrophotometer used the absorption of light by these elements to measure their concentration. The sampling modes were as follows: optical system—two-beam, monochromator—Cherny–Turner, spectral range—190–900 nm, spectral gap—0.2, 0.7 nm (manual switching), background corrector—deuterium, and simultaneous switching on (one works, the other is heating up). Nozzles: flame, hydride, or mercury attachments. Flame atomizer—titanium burner, Pt/Ir capillary, ceramic impactor, corrosion resistant atomizer, high-temperature C_2_H_2_—N_2_O flame burner.

The scientific novelty of this research is the new, simpler, and more environmentally friendly method, which was proposed for the production of graphene materials from RH by carbonation and chemical activation.

## 3. Results

### 3.1. Graphene Synthesis and Characteristics

KOH is a known hydroscopic material, which allows carbonization reactions to occur at substantially lower temperatures (its melting point is 380 °C) compared to standard pyrolysis, which is governed mostly by radical processes and, in turn, leads to disproportionate reactions yielding tar formation. The purpose of KOH is, therefore, to provide both carbon retention (higher yields, e.g., a smaller number of volatiles are formed as hydrocarbon tar) and high surface area.

KOH activation is a well-known method to generate the pore network in carbons; the activation mechanism is not yet well understood because of the complexity due to the large number of variables in both the experimental parameters and the reactivity of different precursors used. In general, the interaction of carbon and KOH starts with solid–solid reactions and then proceeds via solid–liquid reactions, including the reduction of potassium (K) compound to form metallic K, oxidation of carbon to carbon oxide and carbonate, and other reactions among various active intermediates [29].

**Figure 2:**
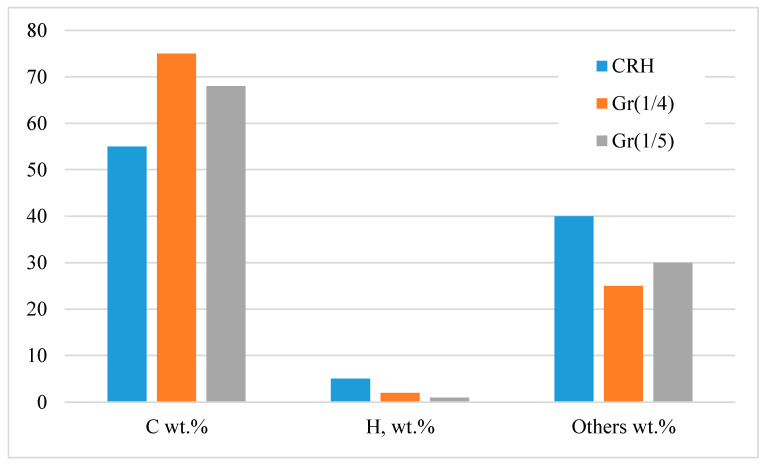
Elemental analysis of CRH, Gr(1/4), and Gr(1/5) samples.

**Figure 3:**
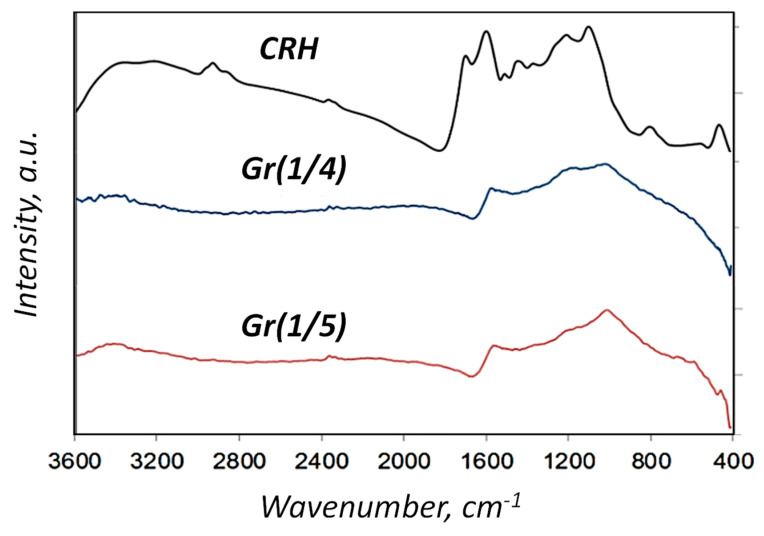
IR spectroscopy of CRH, Gr(1/4), and Gr(1/5) samples.

Carbonation at 1123 K, in combination with chemical activation, showed an increase in carbon content (from ~55 wt.% CRH to ~75 wt.% in Gr(1/4)) and a decrease in hydrogen content (Figure 2). From 25 wt.% to 40 wt.%, there were various substances, such as silicon, potassium, sodium, iron, and others. Therefore, it was necessary to carry out the separation process after chemical activation. This feature was also revealed using infrared spectroscopy (Figure 3). The infrared spectrum of CRH was characterized by different signals attributed to different functional groups: at about 3000 cm^−1^, low-intensity bands were due to stretching vibrations of aliphatic and aromatic C-H bonds, while in the mid-frequency range (1700 and 1000 cm^−1^), a wide combination of peaks was generated based on the overlap of adsorption bands of the carbon skeleton (C=O, C=C, C-C, C-H-C-O stretching and bending modes) [25,27,30]. The Gr(1/4) and Gr(1/5) spectra were characterized by a typical broad shape of large condensed networks of aromatic carbon. Only peaks due to C=C stretching modes and overlapping peaks between 900 and 1600 cm^−1^ due to frame vibrations were detected. The direct current conductivity (σ) of dispersions of solid samples prepared for measurements by IR spectroscopy (0.5–0.8 wt.% in KBr) was: σCRH = 14 ± 3 nS/m, σGr(1/4) = 80 ± 10 nS/m, and σGr(1/5) = 60 ± 10 nS/m. The DC conductivity trend confirmed that a longer conductive portion was established in the samples.

**Figure 4:**
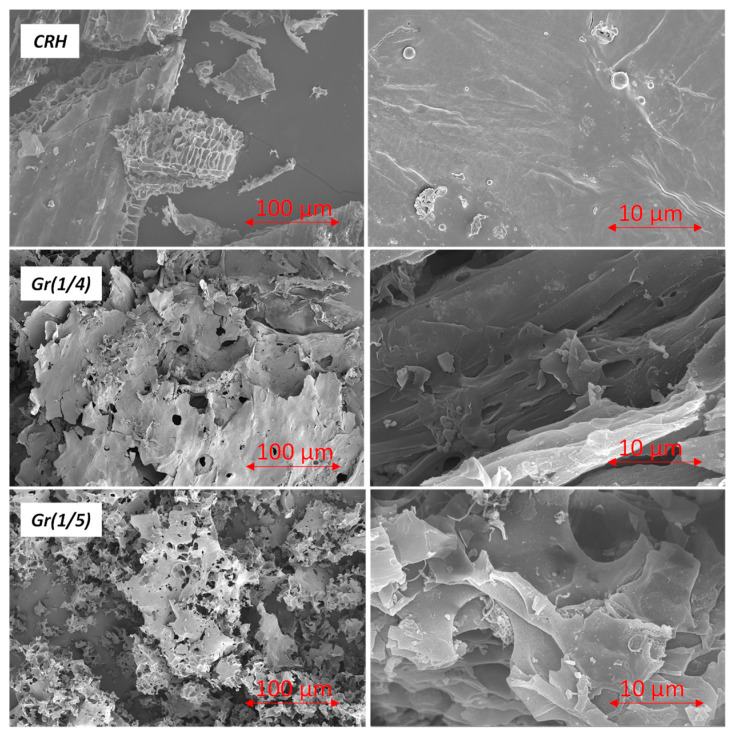
SEM imaging of CRH, Gr(1/4), and Gr(1/5) samples (left side 100 µm and right side 10 µm magnification).

SEM images of CRH, Gr(1/4), and Gr(1/5) are presented in Figure 4 at 100 µm and 10 µm magnifications. CRH is a highly porous material with a large internal surface area, although no significant changes in its morphology compared to raw RH were observed. Samples Gr(1/4) and Gr(1/5) exhibited a layered structure with a crumpled silk veil effect and folded areas. In general, after activation, the morphology of the samples appeared more defined and ordered (Figure 4, right): graphene sheets fold into a layer-by-layer structure over the entire cross-section, continuously expanding in the longitudinal direction. However, during thermal annealing, micro voids are formed between the graphene sheets, resulting in a higher porosity.

**Figure 5:**
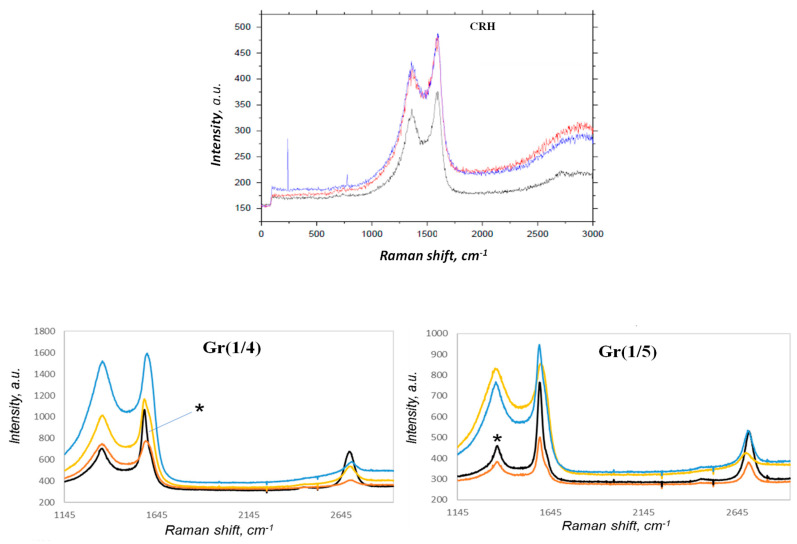
Raman spectra of CRH, Gr(1/4), and Gr(1/5) from different spatial points of the graphene samples.

CRH exhibited Raman features associated only with amorphous carbon (D and G bands), with no trace of the G’ band, namely the band (also called 2D) around 2700 cm^−1^ typical of non-amorphous sp^2^ carbon structures (Figure 5). The Raman spectra of Gr(1/5) and Gr(1/4) in Figure 5 clearly indicate a mixture of amorphous and graphene components. Significant spatial heterogeneity was detected. The figure shows analysis results from different spatial points. However, two main components could be identified: amorphous carbon (with detectable D and G bands around 1352 and 1594 cm^−1^) and graphene (with detectable D, G, and G’ bands).

We analyzed the I_G_/I_G’_ ratio only for those points that were amorphous; Gr was considered negligible (marked * in Figure 5). For both samples Gr(1/5) and Gr(1/4), the I_G_/I_G’_ ratio was close to 1.56 ± 0.10, indicating a multilayer structure. By comparing the Gr(1/4) and Gr(1/5) samples in the limited sampling we performed, it could be estimated that an activation ratio of 1:5/CRH:KOH provided higher graphene content than the 1:4/CRH:KOH ratio.

### 3.2. Graphene Material Synthesis and Characteristics

**Figure 6:**
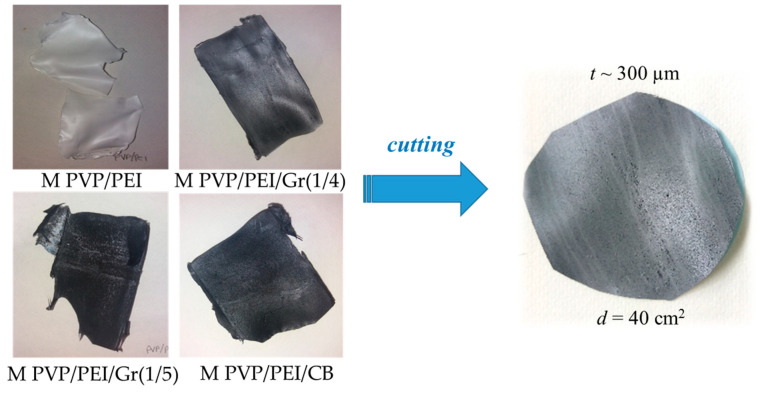
Images of the synthesized graphene membranes.

All the graphene membranes were obtained according to the KOH activation, as previously shown by [29]. An overview of the composition of the filling solution is given in Table 1, and a view of the obtained graphene material is shown in Figure 6. It is clearly seen that the fabricated membranes consisted of a continuous polymer phase, as shown in Figure 6, and likely a dispersed carbon particle phase. The latter would have a loading of about 16% by weight and might be well below the percolation threshold to establish a physically connected continuous phase. Consequently, the function of the carbon particle phase may primarily serve as an adsorbent to entrap the solutes tested. Thus, the membranes’ function might be related to adsorption rather than size exclusion.

The change in cross-sectional morphology of the PEI materials after the addition of modified graphene (washed and functionalized Gr(1/4)) and carbon black addition to the graphene was analyzed using SEM (FEI Inspect™ S50, Thermo Fisher Scientific Inc., Waltham, MA, USA) at «National nanotechnology laboratory of open type» Almaty, Kazakhstan). Before SEM analysis, the dry membrane was cryogenically disrupted in liquid water, and a thin layer of gold was deposited using a sputtering device.

**Figure 7:**
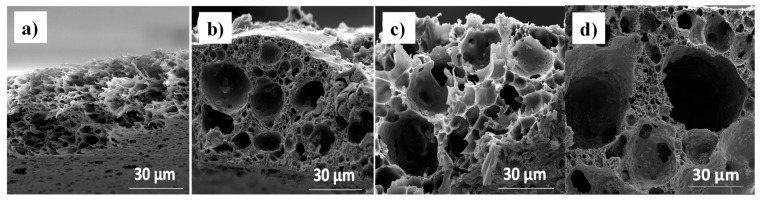
SEM images of samples (**a**) MPVP/PEI, (**b**) MPVP/PEI/Gr(1/4), (**c**) MPVP/PEI/Gr(1/5), and (**d**) MPVP/PEI/CB.

Figure 7 shows SEM images of a cross-section of the composite matrix with different amounts of additive dosage. All samples had an asymmetric structure with a dense top layer followed by a porous sublayer with fully developed macropores, which is a typical morphology of material fabricated by the phase inversion method.

**Figure 8:**
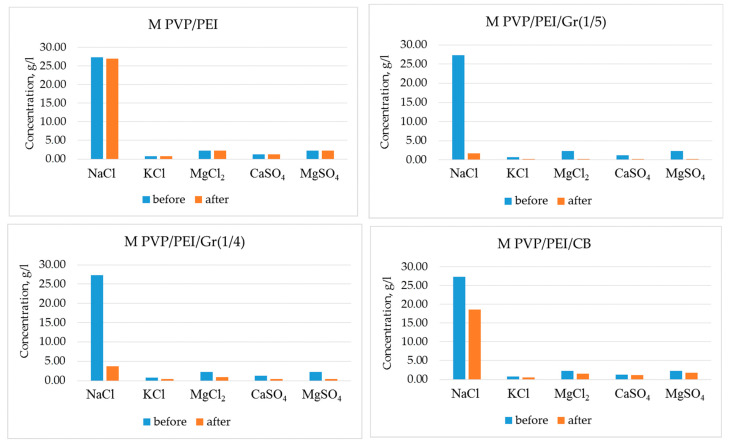
Salt concentration in water before and after desalination with different graphene membranes.

The desalination properties of the different graphene membrane types were tested for NaCl, KCl, MgCl_2_, CaSO_4_, and MgSO_4_ using a calibrated atomic absorption flame emission spectrophotometer. The initial composition of the salt solution (35 g/L) (seawater sample) was as follows: NaCl (78.8%), KCl (2.1%), MgCl_2_ (9.1%), CaSO_4_ (3.5%), and MgSO_4_ (6.5%). The salt concentration before and after filtration is shown in Figure 8. The initial salt concentration was: NaCl—27.3 g/L, KCl—0.7 g/L, MgCl_2_—2.275 g/L, CaSO_4_—1.225 g/L, and MgSO_4_—2.275 g/L. MPVP/PEI/Gr(1/5) was found to be the best graphene filter for desalination. Salt concentrations were reduced to the following values: NaCl—1.65 g/L, KCl—0.189 g/L, MgCl_2_—0.199 g/L, CaSO_4_—0.183 g/L, and MgSO_4_—0.267 g/L.

Based on the above results, we can conclude that graphene materials obtained by the NIPS method showed a higher efficiency in filtering salts and functioning in desalination compared to the control membrane.

## 4. Discussion

### 4.1. Graphene Synthesis and Characteristics

To obtain graphene with a surface suitable for membranes or filters in desalination, the heat treatment parameters in carbonization (pyrolysis) of agricultural biowaste are important [31]. Parameters in the heat-treating of raw materials without access to air are used to optimize the carbon content of the final product. In the present study, the pre-carbonization and carbonization processes were controlled by measuring the density, elemental composition, mechanical properties, and stacking height of the carbon layer surface. The RH was pre-carbonated before carbonization, and the surface area of pre-carbonized samples was investigated using the analytical device “Sorbtometer M” (Al-Farabi Kazakh National University, Almaty, Kazakhstan) using the low-temperature nitrogen adsorption method (BET method). Calculations for the pre-carbonized samples indicate that the sample surface area ranged from 270 to 350 m^2^/g.

In the case of the presence of silicon in the pre-carbonized samples, the use of alkaline agents, such as NaOH or KOH, is a way to obtain mesoporous materials and wash out from the matrix any water-soluble Na or K silicates formed by the reaction:

2MeOH + SiO_2_ → Me_2_SiO_3_ + H_2_O

where Me = Na, K, and SiO_2_ serve as a template for pore formation. Therefore, 1 M NaOH solution was used to obtain porous materials.

After the carbonization of CRH, the specific surface area increased up to Gr(1/4)—1523 m^2^/g; Gr(1/5)—2817 m^2^/g. Specific pore volume values of Gr(1/4) and Gr(1/5) were 1.165 cm^3^/g and 1.587 cm^3^/g, respectively.

The elemental composition of the samples was evaluated after the washing and functionalization (with -SO_3_H) processes. We observed that the carbon content increased slightly in all samples, and hydrogen content decreased after functionalization, while it decreased after functionalization only for pre-carbonized samples. The sample Gr(1/5) after functionalization exhibited the highest amount of carbon (85%).

Raman investigation was performed to evaluate the presence of structured carbon as graphene and estimate the number of layers characterizing the nanostructures. Both activated samples with ratios 1:4/CRH:KOH (Gr(1/4)) and 1:5/CRH:KOH (Gr(1/5)) exhibited D, G, and G’ bands. A significant spatial heterogeneity was detected, but two major components could be isolated: amorphous carbon (with D and G bands around 1352 and 1594 cm^−1^) and graphene (with detectable D, G, and G’ bands). We analyzed the ratio IG/IG’ only for those spots where amorphous C was considered negligible. For both samples Gr(1/5) and Gr(1/4), the ratio I_G_/I_G’_ was close to 1.56 ± 0.10, suggesting a multilayered structure. Nevertheless, all the Raman spectra showing G’ bands exhibited a very symmetric line shape, typical of a 1–2-layer graphene.

According to the FTIR, Gr(1/4) and Gr(1/5) spectra were characterized by the typical broad shape of large condensed aromatic carbon networks. Only the peak due to C=C stretching modes and the overlapped peaks between 900 and 1600 cm^−1^ due to skeleton vibrations were detected. Overall, the FTIR results indicated that the functionalization of 1 h with H_2_SO_4_ did not introduce significant change in the samples, while that performed for 24 h successfully allowed the introduction of sulfonic groups.

### 4.2. Desalination Characteristics

The variation in cross-sectional morphology of graphene obtained by immersion precipitation and carbon black additive into membrane matrix was analyzed by SEM (FEI Inspect™ S50 at «National nanotechnology laboratory of open type» Almaty, Kazakhstan) analysis. Prior to SEM analysis, the dry graphene material was cryogenically fractured in liquid water, and a thin layer of gold was coated using a sputtering apparatus. SEM analysis was conducted on the best-performing membranes from each graphene synthesis method to understand how differences in synthesis method impact the membrane morphology and, therefore, performance. The surface and cross-section morphologies of the membranes influence the permeability and selectivity of the membranes. Therefore, the surface roughness and surface topography were evaluated. Overall, the membranes displayed an asymmetric structure with a dense top layer followed by a porous sublayer with fully developed macropores, which is the typical morphology for graphene-based material.

The desalination properties of the resulting graphene materials were studied by their ability to remove salts (NaCl, KCl, MgCl_2_, CaSO_4_, and MgSO_4_) from saltwater solutions. Analysis of desalinated solutions by atomic absorption flame emission spectrophotometry showed that the efficiency of water purification using MPVP/PEI/Gr(1/4) and MPVP/PEI/Gr(1/5) was 74–95% for the listed salts. In the case of the reference MPVP/PEI/CB, the efficiency did not exceed 50%. As Aghigh et al. [15] reviewed, graphene materials have many advantages in relation to reversed osmosis when it comes to pressure and energy needs. However, most studies are still based on simulations and not on practical results. Some recent results are summarized by Al Faruque et al. [32] and Le et al. [16]. They stated a general efficiency rate for % SAR (sodium adsorption ratio) in the range of 80–96% for desalination by using graphene-based materials. In view of this, the results in this study fall within this general range. However, for the major type of salt (NaCl), the efficiency was close to 95%. Based on this, we can conclude that obtaining graphene material from agricultural waste and using it for the desalination of saltwater is promising and needs further investigation.

## 5. Conclusions

RH carbonation/activation treatment resulted in the formation of a mixture of graphene and amorphous carbon layers. The activation ratio of 1:5 CRH to KOH provided higher graphene content than the 1:4 CRH to KOH ratio, while the number of layers remained unchanged. Treating the samples with H_2_O_2_ indicated an effect on the graphene content and a detachment from the amorphous carbon. The graphene materials were obtained using immersion deposition methods. SEM results showed that the graphene materials have a different microporous structure of ~10 μm and a thickness of about ~200 μm. The desalination properties of the graphene membranes were tested for NaCl, KCl, MgCl_2_, CaSO_4_, and MgSO_4_. As a result, and even though the membranes’ main function might be related more to adsorption rather than size exclusion, it was revealed that, after filtration, the content of these salts decreased by 74–95%. This displays the efficiency of graphene produced from biowaste and a way to include green technologies to improve water security.

## Figures and Tables

**Table 1:** Graphene material composition.

Sample	NMP mL	PEI g	PVP g	Gr(1/4) g	Gr(1/5) g	Carbon Black g	Thickness µm
M PVP/PEI	1.95	0.41	0.1	-	-	-	150
M PVP/PEI/Gr(1/4)	1.95	0.41	0.1	0.1	-	-	360
M PVP/PEI/Gr(1/5)	1.95	0.41	0.1	-	0.1	-	270
M PVP/PEI/CB	1.95	0.41	0.1	-	-	0.1	370

## Data Availability

Data will be supplied upon request from the authors.
